# Yinling Qushi Granule as a therapeutic agent for metabolic syndrome: pharmacological, molecular targets, and pharmacokinetic insights

**DOI:** 10.1007/s44307-025-00065-9

**Published:** 2025-05-30

**Authors:** Linlin Jin, Shaoqin Lin, Yan He, Peibo Li, Xinyan Chen, Weiwei Su, Fei Tan, Chunxiang He, Zhimin Yang, Hao Wu

**Affiliations:** 1 Present Address: School of Life Sciences, Guangdong Engineering & Technology Research Center for Quality and Efficacy Reevaluation of Post-Market Traditional Chinese Medicine, Sun Yat-Sen University, Guangzhou, 510275 People’s Republic of China; 2 Guangdong Provincial Key Laboratory of Plant Stress Biology, State Key Laboratory of Biocontrol, Sun Yat-Sen University, Guangzhou, 510275 People’s Republic of China; 3 State Key Laboratory of Dampness, Syndrome of Chinese Medicine, The Second Affiliated Hospital of Guangzhou University of Chinese Medicine, Guangzhou, 510120 People’s Republic of China; 4 Present Address: Guangdong Provincial Key Laboratory of Clinical Research On Traditional Chinese Medicine Syndrome, Guangzhou, 510120 People’s Republic of China

**Keywords:** Yinling-Qushi Granule, Metabolic syndrome, Hypertension, Dampness syndrome, Lanosterol-triterpenoids

## Abstract

Metabolic Syndrome (MetS) is a globally prevalent condition with unclear underlying mechanisms. Current treatments primarily focus on alleviating individual symptoms, lacking long-term therapeutic strategies that target the disease’s endotypes. In this study, we found that Yinling Qushi Granule (YLQSG), a formulated prescription for treating Dampness Syndrome (DS) in Traditional Chinese Medicine (TCM), effectively attenuated hypertension associated with MetS. After 12 weeks of YLQSG treatment, MetS patients showed significant reduction in systolic and diastolic blood pressure, body mass index (BMI) and glycated hemoglobin (HbA1c). Chemical analysis has identified a total of 96 components of YLQSG, alongside with 27 serum absorbed prototypes and 13 metabolites. In vivo metabolic profiling revealed critical Phase I metabolism including dehydration, demethylation, and oxidation. By integrating network pharmacology and molecular docking, we proposed 11 triterpenoids from the sovereign herb *Poria cocos* (Schw.) Wolf as active constituents of YLQSG. These triterpenoids showed potent binding affinities with AGTR1 and 11β-HSD1, two crucial druggable targets in the renin–angiotensin–aldosterone system. Pharmacokinetics of these 11 lanosterol-type triterpenoids in rat serum indicated their good drug likeness properties. In summary, our findings highlight the potential of YLQSG in treating MetS, particularly hypertension, and identify its active compounds and potential molecular targets, offering a promising alternative strategy for the long-term management of MetS.

**Trial registration**

ChiCTR, ChiCTR2200063506. Registered 9 September 2022, https://www.chictr.org.cn/showproj.html?proj=174653.

## Introduction

Metabolic Syndrome (MetS) is a cluster of modifiable metabolic risk factors that occur simultaneously, including abdominal obesity, insulin resistance, dyslipidemia, and hypertension (Grundy et al. 2005). The prevalence of MetS is estimated to 20–40% of adults globally (Aguilar et al. 2015; Lovre and Mauvais-Jarvis 2015; Neeland et al. 2024), and is associated with a 2.5- to 2.8-fold increased risk of cardiovascular death (Lakka et al. 2002). Despite of this high morbidity, the percentage of MetS patients who are timely diagnosed and properly treated remains notably low. Up to 50% of patients remain undiagnosed (Rydén et al. 2007), and less than one-third of these diagnosed patients received comprehensive five secondary interventions for cardiovascular diseases (Pagidipati et al. 2017). As for therapy, due to the still unclear etiology of MetS (Cornier et al. 2008; Grundy et al. 2005), current treatments of this disease primarily focus on alleviating individual symptom, such as the use of lipid-lowering drugs for hyperlipidemia. However, there is a lack of synergistic combination therapies that targeting the underlying pathogenesis and multiplex of phenotypic symptoms, underlying an urgent need of addressing this syndrome.

Disease is a concept that refers to pathological alterations occurring within the body, which present as a series of phenotypic symptoms. These symptoms are the individual manifestations of a disease, and modern medicine diagnoses a disease based on groups of symptoms (Nogales et al. 2022). In contrast, Traditional Chinese Medicine (TCM) uses a different approach, diagnosing through the identification of unique “TCM Syndrome” (known as “Zheng” in Chinese) combined with pathological phenotypes (Jiang et al. 2012). “Zheng” refers to an internal, systematic state linked to the causal mechanisms of a disease. For instance, diseases with similar symptoms can be categorized into distinct “Zheng” endotypes, based on their unique symptom characteristics and underlying causes. In TCM theory, the “spleen” is responsible for energy transformation and transportation. Its physiological functions primarily involve distributing the food essence and maintaining the body fluid homeostasis (Dou et al. 2021; Zhang et al. 2021). When “spleen” is functionally compromised, the balance of food and biofluid transformation is disrupted (Dou et al. 2021), leading to dampness accumulation in the body, which refers to a commonly-seen TCM Syndrome, the Dampness Syndrome (DS). According to clinical observations, patients with DS often exhibit symptoms of biofluid imbalance, such as swelling tongue and limbs, greasy hair, and shapeless stools (Shang et al. 2022). Additionally, insufficient food transformation may cause dyspepsia and metabolic abnormalities, which would subsequently lead to metabolic disorders (Guo et al. 2025). Studies reported a correlation between hypertriglyceridemia and the scoring of DS (Zhou et al. 2024), implying a potential causative involvement of DS in Metabolic diseases.

TCM treatment is based on both “Zheng” and disease phenotypes. For DS, in particular, TCM interventions involve two major strategies: “transforming dampness in the body” and “repelling dampness from the body”, with the latter being particularly noted for its high and fast-acting efficacy. The Fuling-Zexie decoction, first recorded by the Medical Shrine Zhang Zhongjing in the Jinkui Yaolue (Essentials of the Golden Chamber), is a classical TCM formula used for dampness elimination (Lu et al. 2024). The Yinling Qushi Granule (YLQSG) is a modern TCM prescription modified from the Fuling-Zexie Decoction. By removing *Alisma orientale* (Sam.) Juzep. (Zexie) and adding herb like *Polyporus umbellatus* (Pers.) Fries (Zhuling), *Artemisia capillaris* Thunb. (Yinchen), and *Glehnia littoralis* Fr.Schmidt ex Miq. (Beishashen), YLQSG enhances spleen-tonifying effects while sustaining its original dampness-resolving properties (Wu et al. 2014; L. Yang et al. 2021). However, it is unknown whether this prescription is beneficial for patients of DS and metabolic diseases like MetS. And if so, what’s its pharmacological characteristics, material basis, and potential mechanisms of actions?

Hence, in this study, we aimed of investigating the clinical efficacy of YLQSG, in both treating DS and attenuating metabolic disruptions in MetS patients. Not limited to efficacy, we went further to explore its chemical composition, bioactive constituents contributing to its effects, in vivo absorption and metabolism profiling, pharmacokinetic behaviors, network of actions, and potential molecular targets comprehensively, which shed light on the great potential of YLQSG as a promising therapeutic intervention of MetS.

## Materials and methods

### Human participants

This study was registered at China Clinical Trial Registration Center (ChiCTR) (registration No. ChiCTR2200063506) and ethical approved by the Ethics Review Board of the Second Affiliated Hospital of Guangzhou University of Chinese Medicine (Approval No. BF- 2022–086 - 01). All participants gave written informed consent prior to enrollment.

A total of 60 volunteers (male = 27; female = 33; mean age 47.00 ± 11.46 years) diagnosed with MetS were recruited. The diagnosis of MetS was based on the Chinese Guidelines for the Prevention and Control of Type 2 Diabetes Mellitus (2020 edition), meeting three out of five criteria: (1) Abdominal obesity: waist ≥ 90 cm for males and ≥ 85 cm for females; (2) Hyperglycemia: fasting blood glucose (FBG) ≥ 6.1 mmol/L, 2-h post-glucose load with blood glucose ≥ 7.8 mmol/L, or a prior diagnosis of diabetes under treatment; (3) Hypertension: blood pressure ≥ 130/85 mmHg or a diagnosis of hypertension; (4) Fasting blood TG ≥ 1.70 mmol/L; and (5) Fasting HDL-C < 1.04 mmol/L.

DS was assessed using the DS Assessment Scale, developed with reference to the DS Assessment Operating Procedure (T/CACM 1388–2022) issued by the Chinese Society of Traditional Chinese Medicine. The degree of dampness was evaluated by 30 phenotype indicators, on a scoring scale of 0–4 depends on severity, by experienced TCM practitioners.

Participants meeting any of the following conditions were excluded: (1) diseases known to cause secondary disruption of glucose/lipid metabolism and blood pressure, including: nephrotic syndrome, hypothyroidism, renal failure, liver diseases, systemic lupus erythematosus, myeloma, glycogen storage disorder, lipoatrophy, acute porphyria, or polycystic ovary syndrome; (2) medication induced secondary disruptions, including phenothiazines, beta-blockers, or adrenal corticosteroids; (3) persistent hypertension after treatment: SBP ≥ 180 mmHg or DBP ≥ 110 mmHg; (4) FBG ≥ 11.1 mmol/L, under treatment; (5) persistent dyslipidemia after treatment: TC ≥ 6.22 mmol/L, LDL-C ≥ 4.92 mmol/L, or TG ≥ 3.39 mmol/L; (6) severe comorbidities, including tumor malignancies, severe cerebrovascular or cardiac accidences, hematologic diseases, trauma, or recent major surgeries; (7) pregnancy, lactation, or plans for pregnancy during the study (including participant’s spouse); (8) a history of drug allergies; (9) patients with mental disorders; (10) conditions deemed incompatible with long-term participation in the study.

### Clinical study design and procedures

YLQSG (Lot No. 2207321) was manufactured by Jiangyin Tianjiang Pharmaceutical Co., Ltd. (Wuxi, China) with GMP certification. Participants took one dose of YLQSG daily, preferably post-meal, for continuous 12 weeks. Body physical measurements, blood biochemical assays, and DS assessments were performed at three time points: baseline (week 0), mid-study (week 4), and study endpoint (week 12), respectively.

### Materials and reagents

Reference herbs *Poria cocos* (Schw.) Wolf (Fuling, FL), *Atractylodes macrocephala* Koidz. (Baizhu, BZ), *Zingiber officinale* Rosc. (Ganjiang, GJ), *Polyporus umbellatus* (Pers.) Fries (Zhuling, ZL), *Cinnamomum cassia* Presl (Guizhi, GZ), *Artemisia capillaris* Thunb. (Yinchen, YC), *Glehnia littoralis* Fr.Schmidt ex Miq. (Beishashen, BSS), *Glycyrrhiza uralensis* Fisch. (Gancao, GC) were provided by the Second Affiliated Hospital of Guangzhou University of Chinese Medicine, and had been authenticated by Prof. Wenbo Liao from Guangdong Key Laboratory of Plant Resources, School of Life Sciences, Sun Yat-sen University.

MS grade methanol and acetonitrile were obtained from Fisher Scientific Inc. (Fair Lawn, NJ, USA). MS grade formic acid was purchased from Sigma-Aldrich (St. Louis, MO, USA). Milli-Q distilled water was freshly purified by reverse-osmosis system (Merck Millipore, Germany) before use. HPLC grade ethyl acetate was purchased from Damao Chemical Reagent Factory (Purity ≥ 99.5%, Tianjin, China). Stable isotope labeled Myristic acid-D27 (Cambridge Isotope Laboratories, Inc., Andover, MA, USA) was used as internal control.

### Animals

SPF grade Sprague–Dawley rats (*n* = 6, male, 8–10-week-old, 300 ± 20 g) were obtained from the Guangdong Medical Laboratory Animal Center (Guangzhou, China, Certificate No. SCXK 2022–0002), maintained in environmental conditions with a temperature of 20–25 °C, humidity of 55 ± 15%, and a 12-h light/dark cycle. Animals were acclimated for 7 days prior to drug administration. The experimental procedures and protocols have been approved by the Animal Ethics Committee of the School of Life Sciences, Sun Yat-sen University (Ethics approval No. SYSU-lS-IACUC- 2024–0130, Guangzhou, China), with every effort being made to minimize the suffering of the animals.

### Chemical profiling of YLQSG

5.0 g of YLQSG (Jiangyin Tianjiang Pharmaceutical Co., Ltd, Lot No. 2207321) was weighted, dissolved in 10 mL of deionized water, ultrasonic extracted for 10 min, and filtered through 0.22 μm membrane. 10 μL of filtrate was injected into an ultra-fast liquid chromatography quadrupole time-of-flight tandem mass spectrometry (UFLC-Q-TOF–MS/MS) (Triple TOF™ 5600 plus, Sciex, Framingham, MA, USA) analysis for chemical characterization.

To Prepare working solution of each single herb, 4.8 g of *Poria cocos* (Schw.) Wolf (Fuling, FL), 1.8 g of *Atractylodes macrocephala* Koidz. (Baizhu, BZ), 0.8 g of *Zingiber officinale* Rosc. (Ganjiang, GJ), 1.2 g of *Polyporus umbellatus* (Pers.) Fries (Zhuling, ZL), 1.2 g of *Cinnamomum cassia* Presl (Guizhi, GZ), 1.5 g of *Artemisia capillaris* Thunb. (Yinchen, YC), 1.2 g of *Glehnia littoralis* Fr.Schmidt ex Miq. (Beishashen, BSS) and 1.2 g of *Glycyrrhiza uralensis* Fisch. (Gancao, GC) were weighed, and extracted respectively with 100 mL boiling water for 1 h, twice. The aqueous extracts from two extractions were combined, filtered, and concentrated to a total volume of 5 mL. 10 μL of each extraction was injected into the UFLC-Q-TOF–MS/MS system for ingredient attribution analysis.

For chemical profiling, the chromatographic separation was carried out by a Kinetex C18 column (3 × 150 mm, 2.6 μm, Phenomenex, USA) with mobile phase system composed of A (0.1% formic acid aqueous solution, *v/v*) and B (acetonitrile). The elution was conducted at 0.3 mL/min flow rate, 40 ℃ column temperature for a total of 38 min, as listed below: 5–30% B (0–20 min); 30–95% B (20–27 min), maintained at 95% B (27–32 min); 95–5% B (32–33 min); and 5% B for 5 min to equilibrate the system (33–38 min).

The effluent from the UFLC was introduced into a TripleTOF 5600 Plus mass spectrometer (AB Sciex, USA) by electrospray ionization (ESI). The main parameters of mass spectrometry were as follows: ion source gas 1 and gas 2 were both 55 psi, curtain gas was 35 psi, ion source temperature was 550 °C, ion spray voltage floating was 5,500 V in positive mode while − 4,500 V in negative mode, collision energy was 35 V, collision energy spread was 15 eV, and declustering potential was 80 V. Nitrogen was used as nebulizer and auxiliary gas. Samples were analyzed in both positive and negative ionization modes with scanning mas-to-charge (m/z) range from 50 to 1,500 Da. Data were collected (Analyst®, Sciex, Framingham, MA, USA) in information dependent acquisition (IDA) mode.

Post-acquisition data analysis was conducted by PeakView software (version 2.2, AB Sciex, Foster City, USA). Compound annotation was based on chromatographic elution time, elemental composition, fragmentation patterns, and spectral library matches against the Natural Products HR-MS/MS Spectral Library (Natural Products HR-MS/MS Spectral Library, Version 1.0, AB Sciex, Forster City, USA), Metabolite Pilot software (Version 1.0, Sciex, Framingham, MA, USA) and comparison with authentic reference standards where available.

### Drug administration and sampling

In order to investigate the absorbable components in vivo, Yinling Qushi (YLQS) extract was prepared as follows: 77.76 g of *Poria cocos* (Schw.) Wolf (Fuling, FL), 29.16 g of *Atractylodes macrocephala* Koidz. (Baizhu, BZ), 12.96 g of *Zingiber officinale* Rosc. (Ganjiang, GJ), 19.44 g of *Polyporus umbellatus* (Pers.) Fries (Zhuling, ZL), 19.44 g of *Cinnamomum cassia* Presl (Guizhi, GZ), 24.30 g *Artemisia capillaris* Thunb. (Yinchen, YC), 19.44 g *Glehnia littoralis* Fr.Schmidt ex Miq. (Beishashen, BSS) and 19.44 g of *Glycyrrhiza uralensis* Fisch. (Gancao, GC) were weighted. The weighted herbs were mixed thoroughly and extracted with 750 mL boiling water for 1 h, twice. The aqueous extracts were combined, filtered, and concentrated by rotary evaporation to a total volume of 12 mL, with a final extraction yield of 18.5 g crude herbal materials per 1 mL of Yinling Qushi (YLQS) extract.

SD Rats were fasted for 12 h, followed by orally administrated with Yinling Qushi (YLQS) extract at a dose of 36.99 g/kg (6.7 mL/kg). 200 μL of venous blood was collected at 0 min, 15 min, 30 min, 1 h, 2 h, 4 h, 6 h, 8 h, 10 h, 12 h, and 24 h post-gavage. Serum samples were freshly prepared by centrifugation (3,000 g, 20 min, 4 ℃), supernatant collection and storage at − 80 ℃ till analysis.

For serum extraction, 90 μL serum of indicated time points were pre-mixed with 10 μL IS working solution (myristic acid-D27, 10 μg/mL in methanol). 1 mL ethyl acetate was added to 100 μL premixed serum, vortexed vigorously for 5 min, and centrifuged (10,000 g, 15 min, 4 ℃) for protein precipitation. 700 μL of supernatant was transferred and evaporated to dryness under nitrogen flow. Prior to assay, samples were re-suspended with 100 μL methanol: distill water solution (4:1, v/v), sonicated and vortex-mixing for dissolve completely, and centrifuged (10,000 g, 20 min, 4 ℃). 10 μL of supernatant was analyzed using a connected system of AQUITY UPLC (Waters Corp., Milford, MA, USA) hybrid ZenoTOF 7600-MS/MS system (AB Sciex, Framingham, MA, USA) with electrospray ionization (ESI) source.

### Analysis of absorbable components of Yinling Qushi (YLQS) extract

A reversed-phase Kinetex C18 column (2.1 × 100 mm, 2.6 μm, Phenomenex, CA, USA) was used, with 0.3 mL/min flow rate at 40 ℃. The mobile phase consisted of an aqueous solvent A (0.1% formic acid, *v/v*) and organic solvent B (acetonitrile with 0.1% formic acid, *v/v*). The elution gradient was as follows: 5–30% B (0–20 min); 30–95% B (20–27 min), maintained at 95% B (27–32 min); 95–5% B (32–33 min); and 5% B (33–38 min) to equilibrate the system.

The instrumental parameters of mass spectrometry were as follows: ion source gas 1 and gas 2 were both 55 psi, curtain gas was 35 psi, the CAD gas was 8 psi, and the ion source temperature was 550 °C. The ion spray voltage floating (ISVF) was set to 5500 V for positive mode and − 4500 V for negative mode. The collision energy (CE) and collision energy spread (CES) were set to 35 V and 15 eV, and declustering potential was 80 V. The mass-to-charge ratio (m/z) range was set to 50–1500 Da, with an accumulation time of 0.15 s. Data were collected by SCIEX OS software (version 3.3.0.12027, AB Sciex, Framingham, MA, USA) in information-dependent acquisition (IDA) mode.

### Network pharmacology

Components of YLQSG were input into the SwissTargetPrediction database (Daina et al. 2019), which predicted molecular targets based on structural characteristics. In the SwissTargetPrediction database, potential targets were selected based on the top 100 ranked predictions under the"probability"category. If fewer than 100 targets were predicted, all available targets were included Disease related genes were collected from Online Mendelian Inheritance in Man (OMIM) (Amberger et al. 2019) and Human Phenotype Ontology (HPO) (Köhler et al. 2021). Disease related genes were searched by imputing key words including “metabolic syndrome”, “hypertension”, “hyperlipidemia”, “hyperglycemia”, “type 2 diabetes”, and “obesity”. The intersection targets between the disease genes and the predicted YLQSG targets were visualized by Venn diagram.

To construct the protein–protein interaction (PPI) network, targets at the intersection of the two datasets were imported into the STRING database (version 12.0) (Szklarczyk et al. 2023) The species was limited to “*Homo sapiens*”, and the confidence was set to ≥ 0.7. The clusters were implemented using the k-means clustering function of STRING. The PPI network was visualized by Cytoscape (version 3.10.3) (Shannon et al. 2003), feature target nodes were screened out based on frequency, and the tendency of protein–ligand interaction increases at these nodes (Yu et al. 2007).

### Molecular docking

The triterpenoid structures were sourced from the PubChem database. The structural models of the angiotensin receptor (AGTR1) and 11-β-hydroxysteroid dehydrogenase (HSD11B1) were downloaded from Protein Data Bank (PDB: 4YAY, 2ILT). These structural models were prepared using PyMol v2.5.2 by removing water molecules and co-crystallized ligands. Semi-flexible docking was performed using AutoDock Vina 1.2.0 (Eberhardt et al. 2021), and the results were visualized with PyMol v2.5.2.

### Data analysis

For single-arm clinical trial, the statistical analysis was conducted by SPSS statistics (version 26.0, IBM Corp., Armonk, NY, USA). Considering the relatively limited sample size (*n* > 50) of this study, the Kolmogorov–Smirnov test was employed to evaluate the normality of data distribution. A paired t-test was used for within-subject comparisons of pre- and post-intervention, when data fits Gaussian distribution. Otherwise, a non-parametric Wilcoxon signed-rank test was applied instead. Data are presented as mean ± SD (standard deviation). *P* < 0.05 was considered statistically significant.

Pharmacokinetic parameters were calculated using Drug and Statistics (DAS) software (Version 3.0, Shanghai University of Chinese Medicine, Shanghai, China) with a non-compartmental statistical analysis model. The peak concentration (C_max_) and the time to reach C_max_ (T_max_) were derived from detected data. Results are expressed as mean ± SEM (standard error). Data was presented by using GraphPad Prism (Version 8.0.2, GraphPad software, La Jolla, CA, USA).

## Results

### 12-week YLQSG treatment alleviates DS in MetS patients

**Fig. 1:**
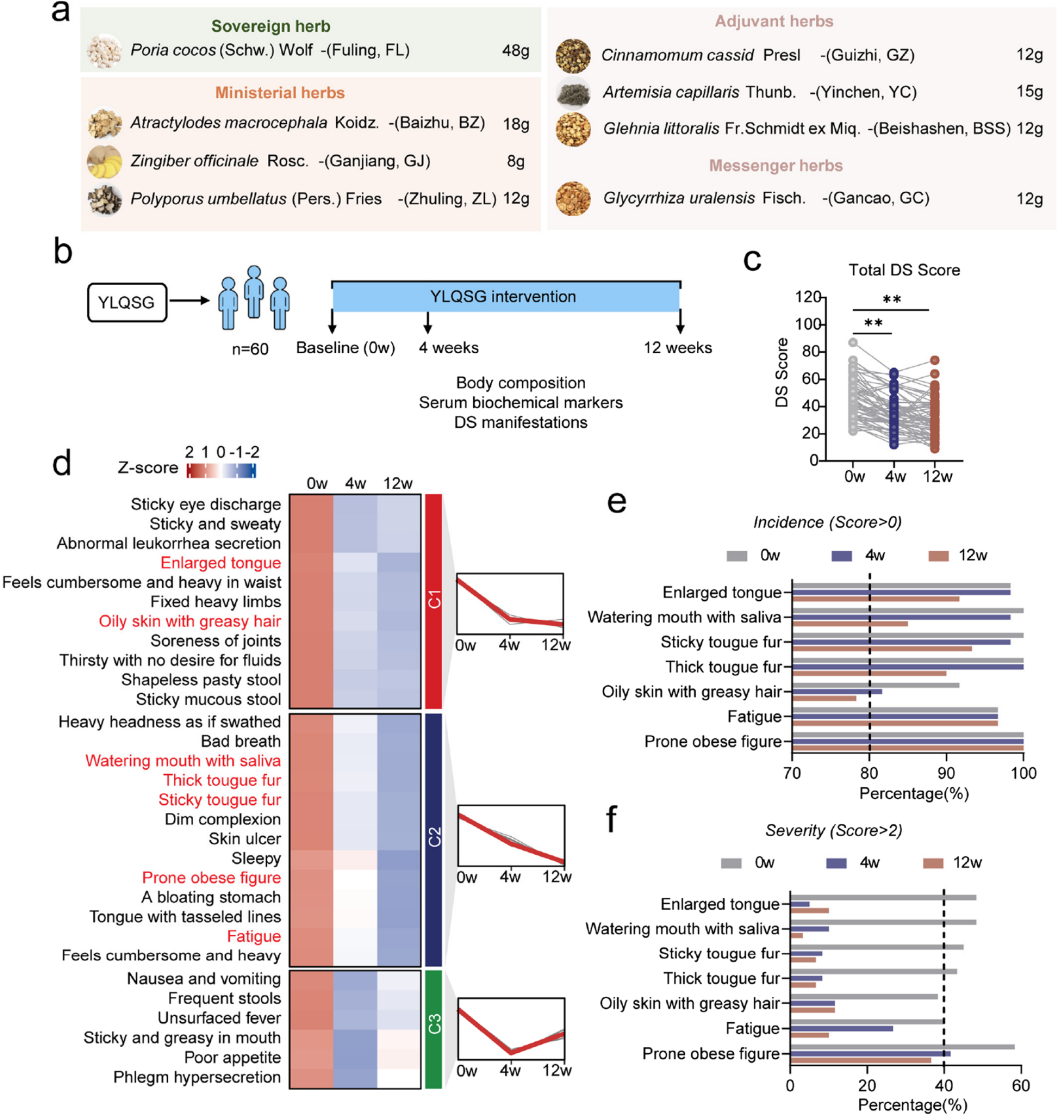
12-week intervention of YLQSG alleviates clinical outcomes of DS in MetS patients (**a**) The formulation of YLQSG comprises eight medicinal herbs, categorized into sovereign, ministerial, adjuvant, and messenger ingredients. **b** The schematic diagram of the clinical study. Participants took one dose of YLQSG daily, preferably post-meal, for continuous 12 weeks. Body physical measurements, blood biochemical assays, and DS assessments were performed at baseline (week 0), mid-study (week 4), and study endpoint (week 12). **c** Total DS scores over the study period (*P < 0.05, ** *P* < 0.01). **d** Changes in DS phenotype scores were analyzed using Fuzzy C-Means Clustering. **e** The incidence and (**f**) severity of specific DS phenotypes at indicated time points. Data were presented as mean ± SD, paired t-test was used to compare pre- and post-intervention outcomes

Our first goal is to investigate the prevalence and degree of DS in MetS patients, and to evaluate the effect of YLQSG (Fig. 1a) in dampness dispelling in participants with metabolic disturbances. To this end, a single-arm clinical study was carried out with 60 MetS patients (Fig. 1b). Baseline characteristics, including body physical health, body composition, and serum biochemical markers, were presented (Table 1). DS was diagnosed and quantitatively evaluated based on a total DS score ≥ 30 by experienced TCM practitioners who were blinded to the patient’s information. At baseline, the mean DS score of participants was 46.68 ± 14.80 (mean ± SD), indicating a high co-occurrence of DS with MetS in this cohort.

**Table 1:** The baseline body composition and serum metabolic features of human participants

Variables	Baseline (*n* = 60)
	**Mean ± SD**
Age (years)	47.00 ± 11.46
Men, n (%)	27, (45.00%)
**Body Measurements**	
Weight(kg)	75.44 ± 11.89
BMI (kg/m^2^)	27.99 ± 3.26
Waist (cm)	94.95 ± 8.26
Hip Circumference (cm)	100.60 ± 14.64
Waist-to-Hip Ratio	0.90 ± 0.13
**Blood Pressure Related**	
Systolic blood pressure (mmHg)	138.30 ± 14.08
Diastolic blood pressure (mmHg)	86.08 ± 10.01
**Blood Lipids Related**	
HDL cholesterol (mmol/L)	1.20 ± 0.35
LDL cholesterol (mmol/L)	3.91 ± 1.12
Total triglycerides (mmol/L)	2.44 ± 1.16
Total cholesterol (mmol/L)	5.71 ± 1.11
**Blood Glucose Related**	
Fasting blood glucose (mmol/L)	5.72 ± 1.26
Fasting insulin (mmol/L)	105.60 ± 61.30
Glycated Hemoglobin (%)	6.02 ± 0.80

*BMI* body mass index, *HDL* high-density lipoprotein, *LDL* low-density lipoprotein

After 4 weeks of YLQSG treatment, the DS score significantly decreased to 35.57 ± 12.87. By the end of 12-week intervention, the DS score further decreased to 31.42 ± 14.46, approaching the cut-off threshold between DS and non-DS (Fig. 1c). A clustering analysis of the 30 DS phenotypes revealed that 24 phenotypes showed remarkable improvement post-treatment. Seven phenotypes, with baseline incidence > 80% and severity > 40%, were significantly ameliorated (Fig. 1d). Notably, after 4 weeks of administration, phenotypes in Cluster 1 showed substantial reductions in both incidence and severity. For example, the incidence of phenotype “oily skin with greasy hair” dropped from 91.67% to 81.67%. While for edema-associated phenotypes like “enlarged tongue” and “watering mouth with saliva”, the severities declined from 48.33% to below 10% (Fig. 1e–f). These together suggested that YLQSG is highly effective in relieving DS in MetS patients.

### YLQSG treatment attenuated hypertension and metabolic disruptions in MetS patients

**Fig. 2:**
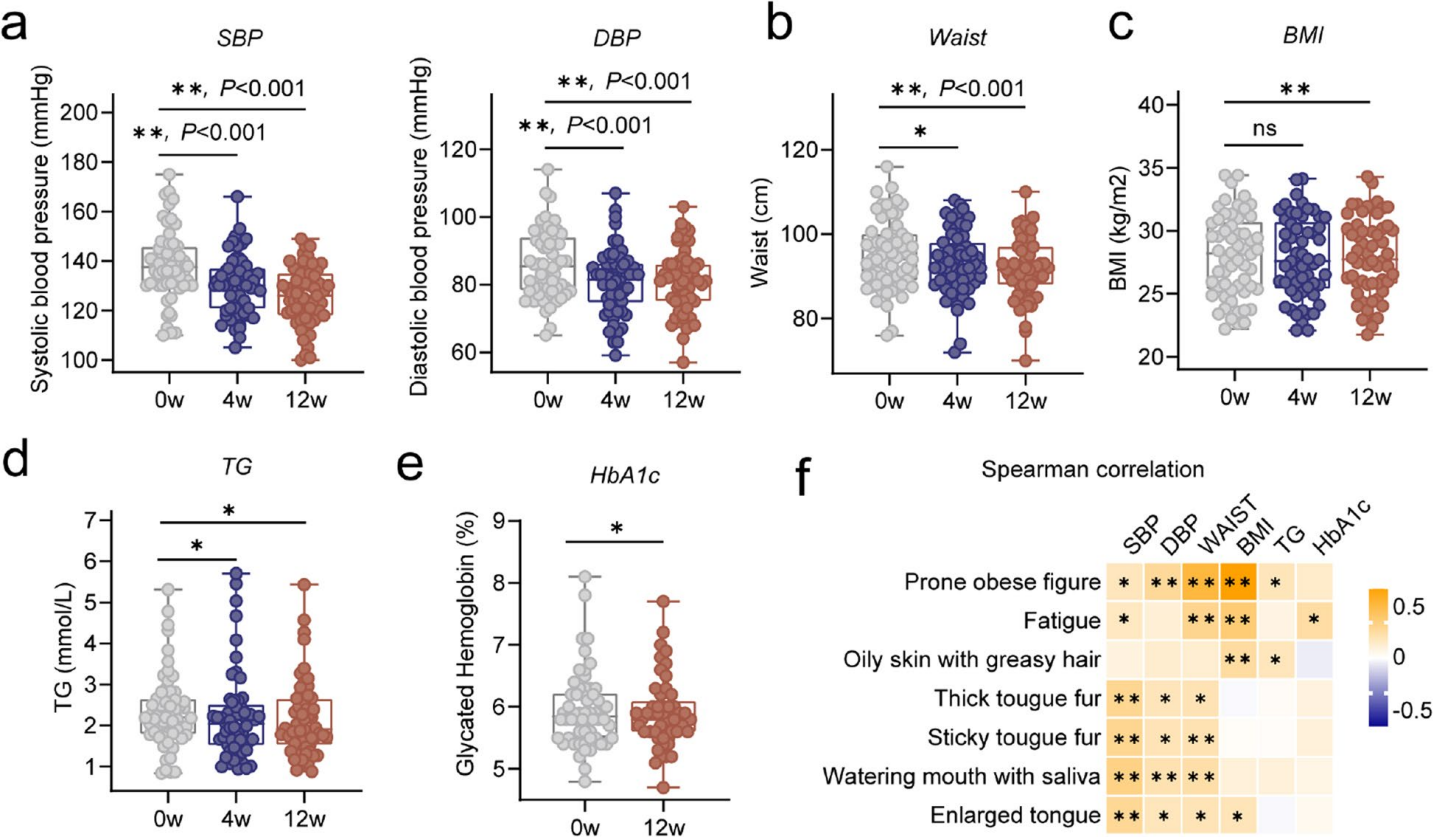
12-week intervention of YLQSG ameliorates hypertension and metabolic disruptions in MetS patients (**a**) Changes in systolic blood pressure (SBP) and diastolic blood pressure (DBP), **b** waist circumference, **c** body mass index (BMI), **d** serum TG, and (**e**) HbA1c levels at 0, 4, and 12 weeks (*P < 0.05, **P < 0.01). **f** Spearman correlation analysis between DS phenotypes and clinical parameters (SBP, DBP, waist circumference, and HbA1c). Data were presented as min to max. Within-subject comparisons were performed using paired t-tests if data were normally distributed (Kolmogorov–Smirnov test, *P* > 0.05); otherwise, the Wilcoxon signed-rank test was used

Having confirmed the efficacy of YLQSG in treating DS, we next evaluated its effects on MetS. At baseline, the participants had a mean systolic blood pressure (SBP) of 138.30 ± 14.08 mmHg and diastolic blood pressure (DBP) of 86.08 ± 10.01 mmHg. After 4 weeks of YLQSG treatment, blood pressure significantly decreased, with SBP reducing to 129.90 ± 12.05 mmHg and DBP to 80.86 ± 9.80 mmHg, approaching the threshold for non-hypertension (Fig. 2a). Not limited to blood pressure regulation, YLQSG intervention also led to a significant reduction in MetS associated obesity, as evidenced by decreases in both waist circumference and body mass index (BMI) (Fig. 2b-c). Reductions in serum triglycerides (TG) and glycated hemoglobin (HbA1c) further indicated improvements in lipid and glucose metabolic disruptions, when compared to baseline (Fig. 2d-e). Spearman correlation revealed strong associations between blood pressure, waist circumference, BMI, and dampness-related phenotypes such as a “prone obese figure” and edema-related symptoms “enlarged tongue”, and “watering mouth with saliva”, which may partially explain the frequent co-occurrence of DS and MetS (Fig. 2f). In summary, these results suggest that YLQSG alleviates both DS and MetS, with a particular emphasis on MetS-associated hypertension and obesity.

### Comprehensive chemical profiling of YLQSG

**Fig. 3:**
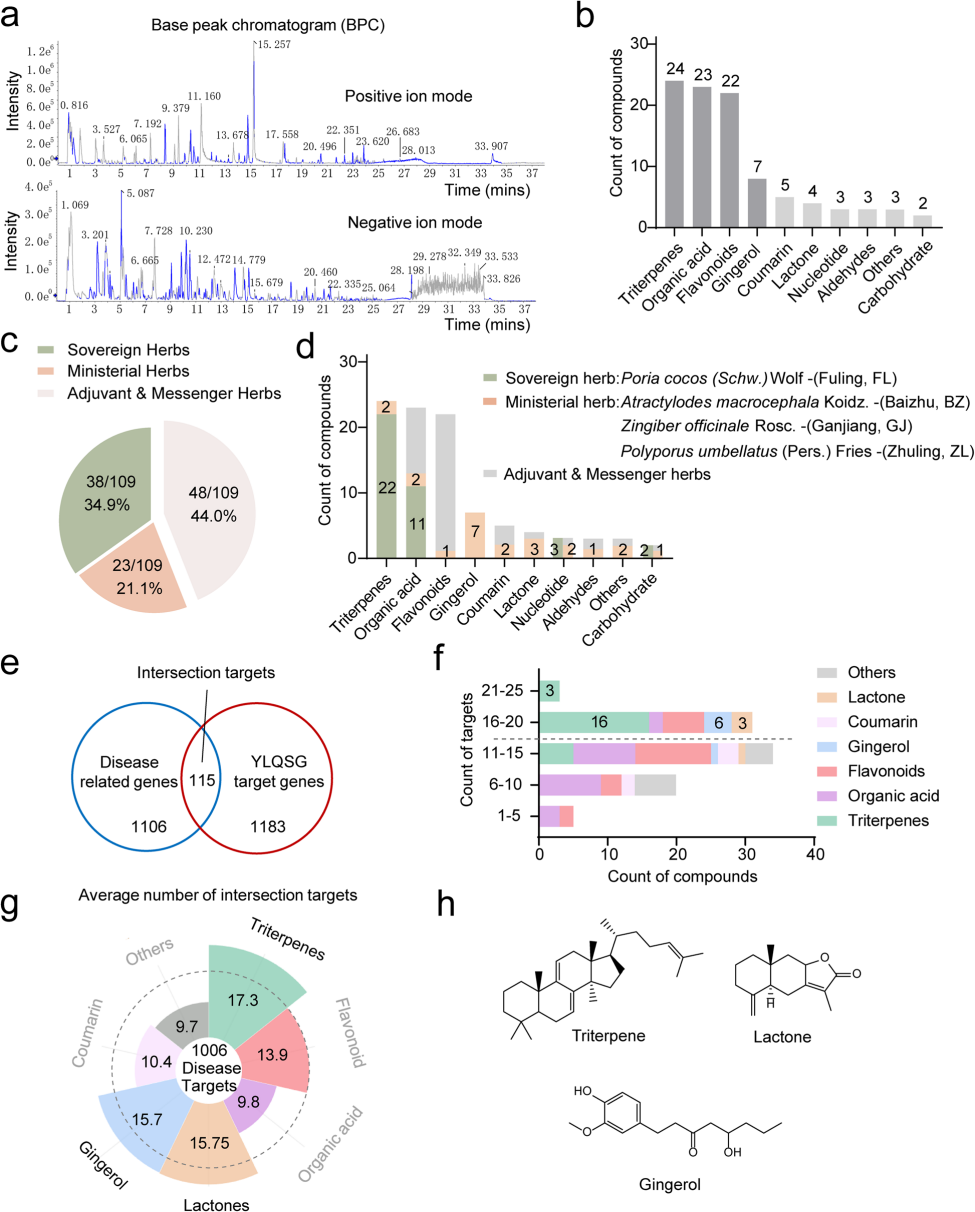
Chemical profiling of YLQSG using UFLC-Q-TOF–MS/MS (**a**) Base peak chromatogram (BPC) of YLQSG in positive and negative ion modes. **b** A total of 96 natural products were identified and categorized into 10 structural classes based on structural skeleton. **c** Proportions of compounds identified in sovereign and ministerial herbs. **d** Structural classifications of compounds derived from the sovereign and ministerial herbs. **f** Herbal attribution to each structural class. **e**–**h** Network pharmacology analysis of YLQSG against MetS: **e** identification of drug-disease intersection targets, **f** frequency distribution of these targets, and (**g**) average frequency of compound targets within each structural class. **h** Representative skeletons of lanosterol triterpenoids, gingerols, and lactones

Given the potent clinical outcomes of YLQSG, we next sought to identify the active constituents responsible for such therapeutic effects. To achieve this, we performed chemical profiling of YLQSG using UFLC-Q-TOF–MS/MS with a total of 96 compounds identified or tentatively characterized (Fig. 3a). These compounds were classified into 10 structural categories, with the majority consisting of 24 triterpenoids, 23 flavonoids, 22 organic acids, and 7 gingerols, collectively accounting for 79% (76/96) of the total (Fig. 3b).

It’s worth mentioning that, like other TCM prescriptions, YLQSG is formulated based on the principles of sovereign, minister, adjuvant and messenger herbs, with sovereign herb playing the most predominant roles, followed by ministerial herbs, and then came adjuvant and messenger herbs. This underscores that for chemical profiling of TCM, it’s far beyond numbers: bioactive compounds from sovereign and ministerial ingredients should overweight and be prioritized. Of the 96 compounds identified, 60.42% (58/96) were derived from sovereign and ministerial herbs (Fig. 3c), indicating the superb of this method with a comprehensive coverage of the key components from feature ingredients. The sovereign herb, *Poria cocos* (Schw.) Wolf (Fuling, FL), predominantly contributed triterpenoids with 22 out of 24 triterpenoids attributed (Fig. 3d). Ministerial herbs contributed compounds such as lactones from *Atractylodes macrocephala* Koidz. (Baizhu, BZ), gingerols from *Zingiber officinale* Rosc. (Ganjiang, GJ), and triterpenoids from *Polyporus umbellatus* (Pers.) Fries (Zhuling, ZL) (Fig. 3d). Detailed information, including retention time, mass-to-charge ratios, formula, and fragmentation ions were listed in supplementary table S1.

To screen out the potential bioactive compounds, we conducted network pharmacology analysis. A total of 115 intersecting targets between MetS and predicted drug targets were identified (Fig. 3e), which each compound contributing between 1 and 25 intersecting targets (Fig. 3f). Compounds with more than 16 intersection targets were predominantly triterpenoids from *Poria cocos* (Schw.) Wolf (Fuling, FL) and *Polyporus umbellatus* (Pers.) Fries (Zhuling, ZL), gingerols from *Zingiber officinale* Rosc. (Ganjiang, GJ), and lactones from *Atractylodes macrocephala* Koidz. (Baizhu, BZ) (Fig. 3, g-h), suggesting a special eye on these three classes of components warrant further exploration.

### Identification of the YLQSG absorbent prototypes and in vivo metabolites in rat serum

**Fig. 4:**
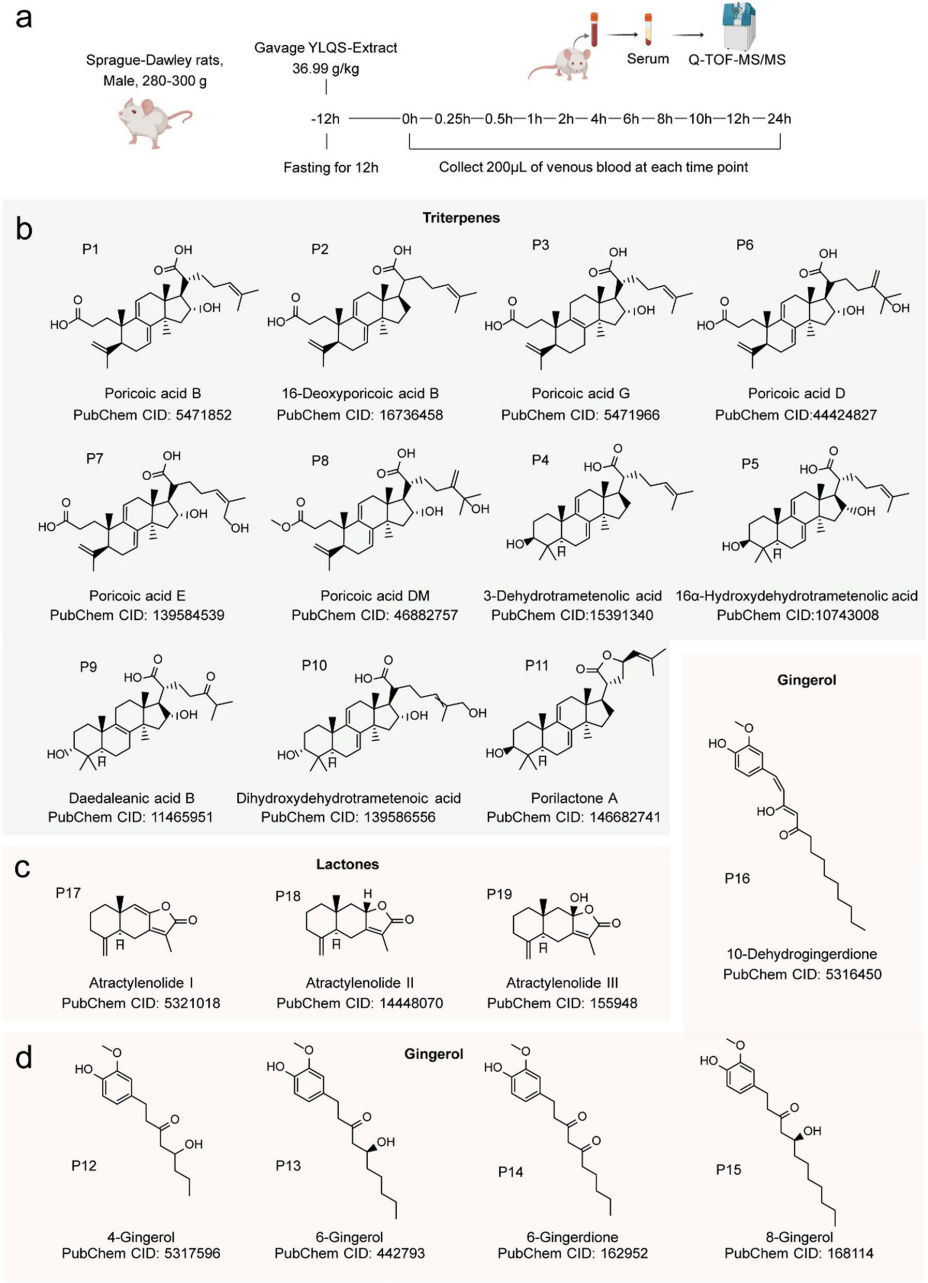
Comprehensive profiling of absorbed prototypes and in vivo metabolites of YLQSG (**a**) Schematic diagram of the study. **b**-**d** Identified prototypes of (**b**) triterpenoids, **c** gingerols, and (**d**) lactones in rat serum. A Prototype or its metabolite was considered a true positive only if detected in serum from at least half rats (*n* ≥ 3) at more than three different time points; otherwise, it was excluded from further analysis

For an orally administered drug, effective absorption into the bloodstream and delivery to target tissues are the classical mechanism of actions for achieving pharmacological effects in vivo (Benet 1978). To this end, we explored the absorbent prototypes of YLQSG and their in vivo metabolism (Fig. 4a). A total of 27 prototypes were identified, including 11 triterpenoids, 3 lactones, 5 gingerols (Fig. 4. b-d), 5 flavonoids, 2 organic acids, and 1 coumarin (Table 2.).

**Table 2.** Identifications of absorbed prototype and in vivo metabolites in rat serum after oral administration of YLQS

No.	Metabolic	RT/min	Formula	[M+H]^+^	[M-H]^-^	Characteristic Fragment ^a^	
				(Error, ppm)	(Error, ppm)	POS	NEG
P1	Poricoic acid B	24.20	C_30_H_44_O_5_	485.32615	ND	485.3264[M+H]^+^	ND
				0.5		235.1683[M+H-C_15_H_22_O_3_]^+^	
M1-1	Demethylated Poricoic acid B	23.94	C_29_H_42_O_5_	ND	469.29435	ND	469.2972[M-H]^-^
					-2.4		425.3078[M-H-COOH]^-^
M1-2	Oxidized Poricoic acid B	23.71	C_30_H_44_O_6_	501.32107	ND	510.3204[M+H]^+^	ND
				-0.8		251.1798[M+H-C_15_H_22_O_3_]^+^	
P2	16-Deoxyporicoic acid B	23.75	C_30_H_44_O_4_	469.33120	ND	469.3323[M+H]^+^	ND
				-0.8		451.3184[M+H-H_2_O]^+^	
						405.3133[M+H-H_2_O-HCOOH]^+^	
						235.1682[M+H-C_15_H_22_O_2_]^+^	
M2-1	Dehydrated 16-Deoxyporicoic acid B	23.74	C_30_H_42_O_3_	451.32040	ND	451.3169[M+H]^+^	ND
				-0.6		433.3079[M+H-H_2_O]^+^	
						405.3124[M+H-HCOOH]^+^	
						235.1710[M+H-C_15_H_20_O]^+^	
P3	Poricoic acid G	23.80	C_30_H_46_O_5_	487.34166	ND	487.3445[M+H]^+^	ND
				-0.3		251.1619[M+H-C_15_H_24_O_2_]^+^	
M3-1	Demethylated Poricoic acid G	23.39	C_29_H_44_O_5_	473.32590	471.31005	473.3299[M+H]^+^	471.3138[M-H]^-^
				-0.5	-3.3	235.1759[M+H-C_14_H_22_O_3_]^+^	427.3222[M-H-C_2_H_4_O]^-^
P4	3-Dehydrotrametenolic acid	25.82	C_30_H_46_O_3_	455.35197	453.33644	455.3512[M+H]^+^	453.3364[M-H]^-^
				-0.9	-2.2	437.3429[M+H-H_2_O]^+^	435.3200[M-H-H_2_O]^-^
						221.1581[M+H-C_15_H_22_O_2_]^+^	
M4-1	Dehydrated 3-Dehydrotrametenolic acid	25.84	C_30_H_44_O_2_	437.34095	ND	437.3408[M+H]^+^	ND
				-1.1		391.3322[M+H-CO-H_2_O]^+^	
						235.1691[M+H-C_15_H_22_]^+^	
						191.1795[M+H-C_15_H_22_-CO_2_]^+^	
M4-2	Oxidized 3-Dehydrotrametenolic acid	23.81	C_30_H_46_O_5_	487.34181	485.32725	487.3445[M+H]^+^	485.3262[M-H]^-^
				-0.5	-2.1	235.1683[M+H-C_15_H_24_O_3_]^+^	441.3364[M-H-C_2_H_4_O]^-^
P5	16alpha-Hydroxydehydrotrametenolic acid	25.27	C_30_H_46_O_4_	471.34725	469.33274	471.3496[M+H]^+^	469.3489[M-H]^-^
				0.8	0.9	221.1938[M+H-C_15_H_22_O_3_]^+^	425.3491[M-H-COOH]^-^
P6	Poricoic acid D	23.76	C_31_H_46_O_6_	515.33549	ND	515.3368[M+H]^+^	ND
				-2.4		497.2701[M+H-H_2_O]^+^	
M6-1	Reduced Poricoic acid D	25.29	C_31_H_48_O_6_	ND	515.33655	ND	515.3361[M-H]^-^
					-2.4		469.3476[M-H-CO-H_2_O]^-^
P7	Poricoic acid E	23.72	C_30_H_44_O_6_	501.32069	ND	501.3205[M+H]^+^	ND
				-0.7		360.2595[M+H-C_23_H_35_O_3_]^+^	
P8	Poricoic acid DM	24.17	C_32_H_48_O_6_	529.35230	ND	529.3513[M+H]^+^	ND
				-0.1		510.287	
M8-1	Oxidized Poricoic acid DM	25.75	C_32_H_46_O_8_	559.32655	ND	559.3265[M+H]^+^	ND
				-1.1			
P9	Daedaleanic acid B	23.25	C_30_H_48_O_5_	ND	487.34106	ND	487.3461[M-H]^-^
					-3.8		469.3318[M-H-H_2_O]^-^
P10	Dihydroxydehydrotrametenoic acid	23.81	C_30_H_46_O_5_	487.34181	485.32619	487.3445[M+H]^+^	485.3325[M-H]^-^
				-0.5	-2.2		441.3371[M-H-C_2_H_4_O]^-^
P11	Porilactone A	25.28	C_30_H_44_O_3_	453.33647	ND	453.3411[M+H]^+^	ND
				0.3		221.1612[M+H-C_15_H_20_O_2_]^+^	
M11-1	Oxidized Porilactone A	23.81	C_30_H_46_O_5_	487.34181	ND	453.3411[M+H]^+^	ND
				-0.5		237.1460[M+H-C_15_H_22_O_3_]^+^	
M11-2	Demethylated Porilactone A	24.87	C_29_H_42_O_3_	439.32049	ND	439.3166[M+H]^+^	ND
				-0.4		221.1626[M+H-C_14_H_18_O_2_]^+^	
P12	4-Gingerol	22.70	C_15_H_22_O_4_	ND	265.14388	ND	265.1473[M-H]^-^
					-2.5		221.1555[M-H-C_3_H_8_]^-^
							175.1174[M-H-C_5_H_14_O]^-^
							147.8364[M-H-C_6_H_13_O_2_]^-^
P13	6-Gingerol	22.76	C_17_H_26_O_4_	295.19044	293.17487	295.1914[M+H]^+^	293.1794[M-H]^-^
				0.2	-3.3	277.2231[M+H-H_2_O]^+^	221.1563[M-H-C_5_H_12_]^-^
						249.1805[M+H-CO-H_2_O]^+^	177.0912[M-H-C_17_H_16_O]^-^
							148.0549[M-H-C_8_H_17_O_2_]^-^
P14	6-Gingerdione	24.86	C_17_H_24_O_4_	293.17419	291.15967	293.1742[M+H]^+^	291.1595[M-H]^-^
				-1.9	-1.8	190.0130[M+H-C_7_H_17_]^+^	
						121.0300[M+H-C_10_H_18_O_2_]^+^	
M14-1	6-Dehydrogingerdione	23.50	C_17_H_22_O_4_	291.15870	289.14354	291.1587[M+H]^+^	289.1432[M-H]^-^
				-1.3	-3.4		218.0122[M-H-C_5_H_11_]^-^
							190.0189[M-H-C_6_H_11_O]^-^
P15	8-Gingerol	26.72	C_19_H_30_O_4_	323.22091	ND	323.2209[M+H]^+^	ND
				-2.4		305.2109[M+H-H_2_O]^+^	
P16	10-Dehydrogingerdione	26.41	C_21_H_30_O_4_	347.22141	345.20404	347.2214[M+H]^+^	345.2037[M-H]^-^
				-0.8	-9.0	329.2106[M+H-H_2_O]^+^	
						121.0656[M+H-C_13_H_22_O_3_]^+^	
						97.0642[M+H-C_15_H_22_O_3_]^+^	
P17	Atractylenolide I	21.04	C_15_H_18_O_2_	231.13777	ND	231.1392[M+H]^+^	ND
				-0.8		213.1260[M+H-H_2_O]^+^	
						185.1327[M+H-HCOOH]^+^	
						157.1001[M+H-HCOOH-C_2_H_4_]^+^	
M17-1	Oxidized Atractylenolide I	23.55	C_15_H_18_O_3_	247.13254	ND	247.1325[M+H]^+^	ND
				-1.4		205.0686[M+H-C_3_H_6_]^+^	
P18	Atractylenolide II	21.62	C_15_H_20_O_2_	233.15330	ND	233.1533[M+H]^+^	ND
				-1.3		215.1429[M+H-H_2_O]^+^	
						187.1478[M+H-HCOOH]^+^	
						159.1171[M+H-HCOOH-C_2_H_4_]^+^	
P19	Atractylenolide III	22.65	C_15_H_20_O_3_	249.14801	247.13312	249.1481[M+H]^+^	247.1328[M-H]^-^
				-2.0	-3.4	231.1354[M+H-H_2_O]^+^	220.0252
						185.0964[M+H-H_2_O-HCOOH]^+^	190.0171
						157.1011[M+H-H_2_O-HCOOH-C_2_H_4_]^+^	
M19-1	Oxidized Atractylenolide III	21.70	C_15_H_20_O_4_	265.14329	ND	265.1433[M+H]^+^	ND
				-0.6		247.1324[M+H-O]^+^	
						191.0699[M+H-HCOOH-C_2_H_4_]^+^	
P20	Liquiritin	8.63	C_21_H_22_O_9_	ND	417.11854	ND	417.1185[M-H]^-^
					-1.4		255.0670[M-H-C_6_H_10_O_5_]^-^
							135.0089[M-H-C_6_H_10_O_5-_C_7_H_4_O_3_]^-^
							119.0504[M-H-C_6_H_10_O_5_C_8_H_7_O]^-^
P21	Ononin	12.91	C_22_H_22_O_9_	431.13346	ND	431.1335	ND
				-0.5		269.0804[M+H-C_6_H_10_O_5_]^+^	
P22	Formononetin	20.41	C_16_H_12_O_4_	269.08035	ND	269.0803	ND
				-1.8		254.0564[M+H-CH_3_]^+^	
P23	Calycosin	14.39	C_16_H_12_O_5_	285.07547	283.05996	285.0755	283.06
				-1.0	-4.4	270.0532[M+H-CH_3_]^+^	268.0373[M-H-CH_3_]^-^
						242.0574[M+H-C_2_H_4_O]^+^	
P24	5,7-Flavonone	13.46	C_15_H_10_O_4_	255.06483	ND	255.0648	ND
				-1.4		199.0747[M+H-C_2_O_2_]^+^	
						98.9833[M+H-C_8_H_12_O_3_]^+^	
P25	Protocatechuic acid	26.21	C_7_H_6_O_4_	ND	153.01979	ND	153.0198
					3.0		109.2094[M+H-CO_2_]^+^
P26	Isochlorogenic acid A	22.86	C_25_H_24_O_12_	ND	515.11822	ND	515.1182
					-2.5		447.1358[M-H-C_3_O_2_]^-^
P27	5,7-Dihydroxycoumarin	33.08	C_9_H_6_O_4_	ND	177.01947	ND	177.0195
					0.8		102.0087[M-H-C_2_H_3_O_3_]^-^

Among the identified compounds, 3 γ-valerolactone—atractylenolide I (P17), atractylenolide II (P18), and atractylenolide III (P19) —were identified based on their featured fragmentation patterns. In positive ion mode, these compounds (P17, P18, P19) displayed [M + H]^+^ ions at *m/z* 231.1392, 233.1533 and 249.1481 Da, respectively. Their γ-lactone moieties underwent loss of H_2_O and CO, resulting in ring cleavage with the characteristic fragment ions [M + H-H_2_O]^+^ and [M + H-H_2_O-CO]^+^ at M- 18.0132 and M- 46.0065. Moreover, an ethylene group (C₂H₄) was lost from the A-ring, leading to the formation of a five-membered ring, a process of which resemble the McLafferty rearrangement (Fig. S1 d). Unlike others, for atractylenolide III (P19), would undergo dehydration [M + H-H_2_O]^+^ before aforementioned fragmentation (Fig. S1 d).

For gingerols, which belong to the methoxyphenol class, five prototypes were identified following YLQSG administration (Fig. 4d). These gingerols—4-gingerol (P12), 6-gingerol (P13), 6-gingerdione (P14), 8-gingerol (P15), and 10-dehydrogingerdione (P16)—were primarily detected in negative ion mode and showed featured fragmentation with the loss of C₃H₈ (43.9918 Da) and C₅H₁₂ (72.0231 Da) groups from the alkyl chain (Fig. S1e).

The most abundant components identified in rat serum were triterpenoids, predominantly derived from the sovereign herb *Poria cocos* (Schw.) Wolf (Fuling, FL). 11 lanosterol triterpenoids were identified as absorbed prototypes (Fig. 4b), alongside 10 characterized metabolites (Table 2.). These lanosterol triterpenoids mainly divided into 2 types, 3,4-*seco-*lanostane and closed-lanostane, based on whether the A ring retains its cyclic structure. In MS^2^, lanosterol triterpenoids typically detected in negative ion mode, characterized by the cleavage of carboxyl group at C21 on the D-ring branch chain, resulting in [M-COOH]^−^. For 3,4-seco-lanostane types, additional cleavage of CH3 COOH from A-ring are observed, yielding [M-CH_3_CH_2_COOH]^−^ fragmentation ions (Jin et al. 2019; M. Yang et al. 2021). Our results showed the characteristic fragment ions produced by the retro Diels–Alder (RDA) reaction, yielding ion fragmentation of two parts: one encompassing the A- and B-rings, and the other containing the cleaved D-ring (Fig. S1a). This fragmentation was more evident in the positive ion mode, likely due to application of Electron Activated Dissociation (EAD), which can induce fragmentation of higher energy bonds like C–C bond cleavage on C-ring, resulting in richer featured fragmentation patterns compared to traditional Collision-Induced Dissociation (CID) mode. Our results showed this RDA cleavage in both lanosterol types, including 3,4-seco-lanostanes poricoic acid B, 16-deoxyporicoic acid B, and poricoic acid G, yielding fragment [M + H-C_15_H_22_O_3_]^+^ at 235.1683, [M + H-C_15_H_22_O_2_]^+^ at 251.1619 and closed-type lanostane including 16α-hydroxydehydrotrametenolic acid, porilactone A and 3-dehydrotrametenolic acid with fragmentation ions at [M + H-C_15_H_24_O_3_]^+^ and [M + H-C_15_H_24_O_2_]^+^ (Fig. S1b).

**Fig. 5:**
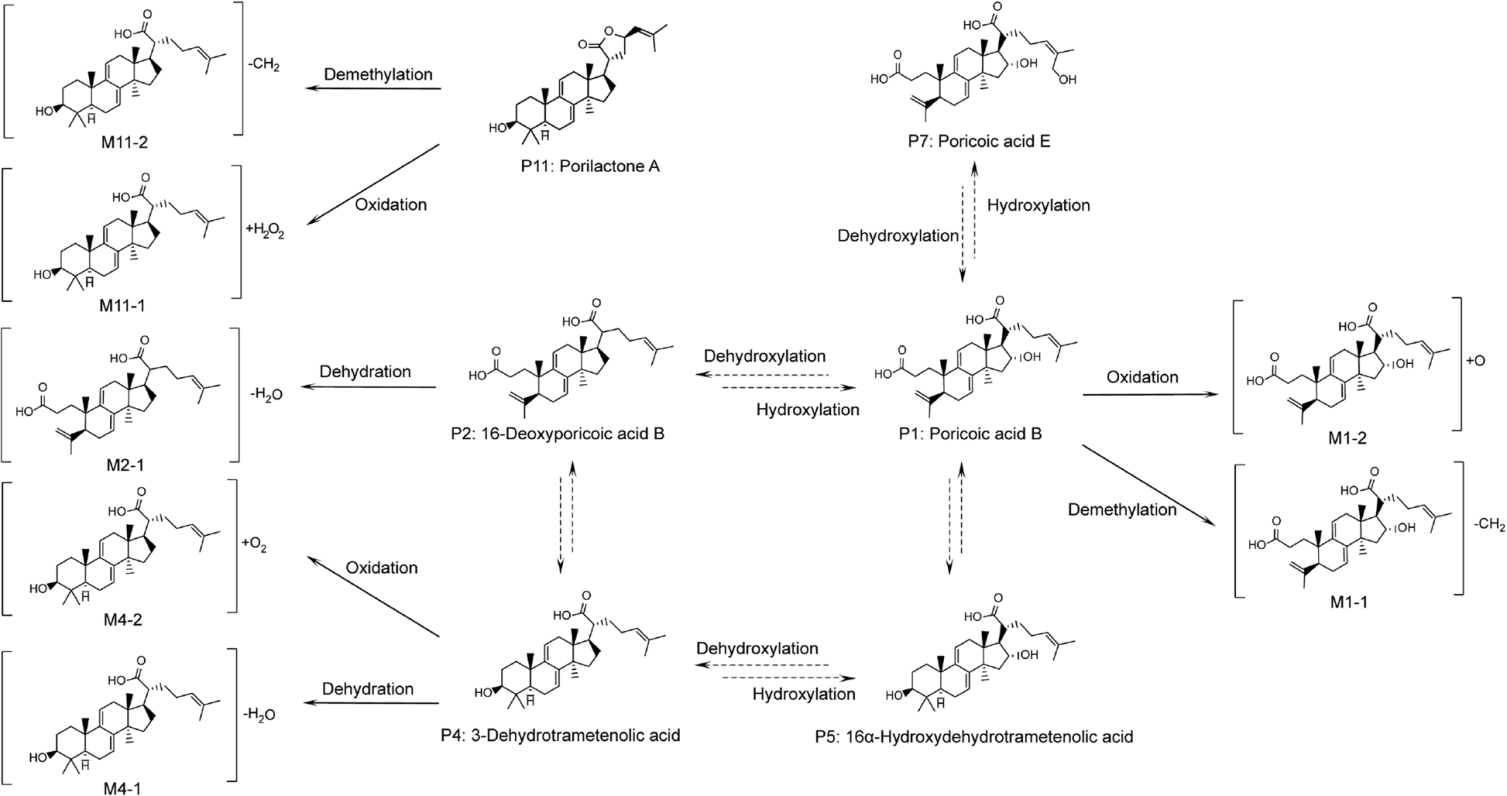
Proposed in vivo metabolism of triterpenoids in SD rats after YLQS extract administration Dashed arrows indicate hypothesized metabolic pathways based on experimental data in this study and previously reported mechanisms concerning the in vivo biotransformation. The number of metabolites in brackets corresponds to that in Table 2.

In vivo, metabolites annotation of lanosterol triterpenoids were achieved by either the previously reported metabolic reactions, or by fully understanding of product ions and fragmentation rules of the parent prototypes. Lanosterol triterpenoids undergo diverse oxidative/deoxidate metabolism (Feng et al. 2018). In line with these, we have identified metabolites of Phase I metabolisms dehydration, oxidation, demethylation (Fig. 5). Detailed information of the metabolites description, chemical composition, retention time (RT), and characteristic product ions of identified compounds is presented (Table 2.).

### Triterpenoids in YLQSG as potential candidates for hypertension by network pharmacology

**Fig. 6:**
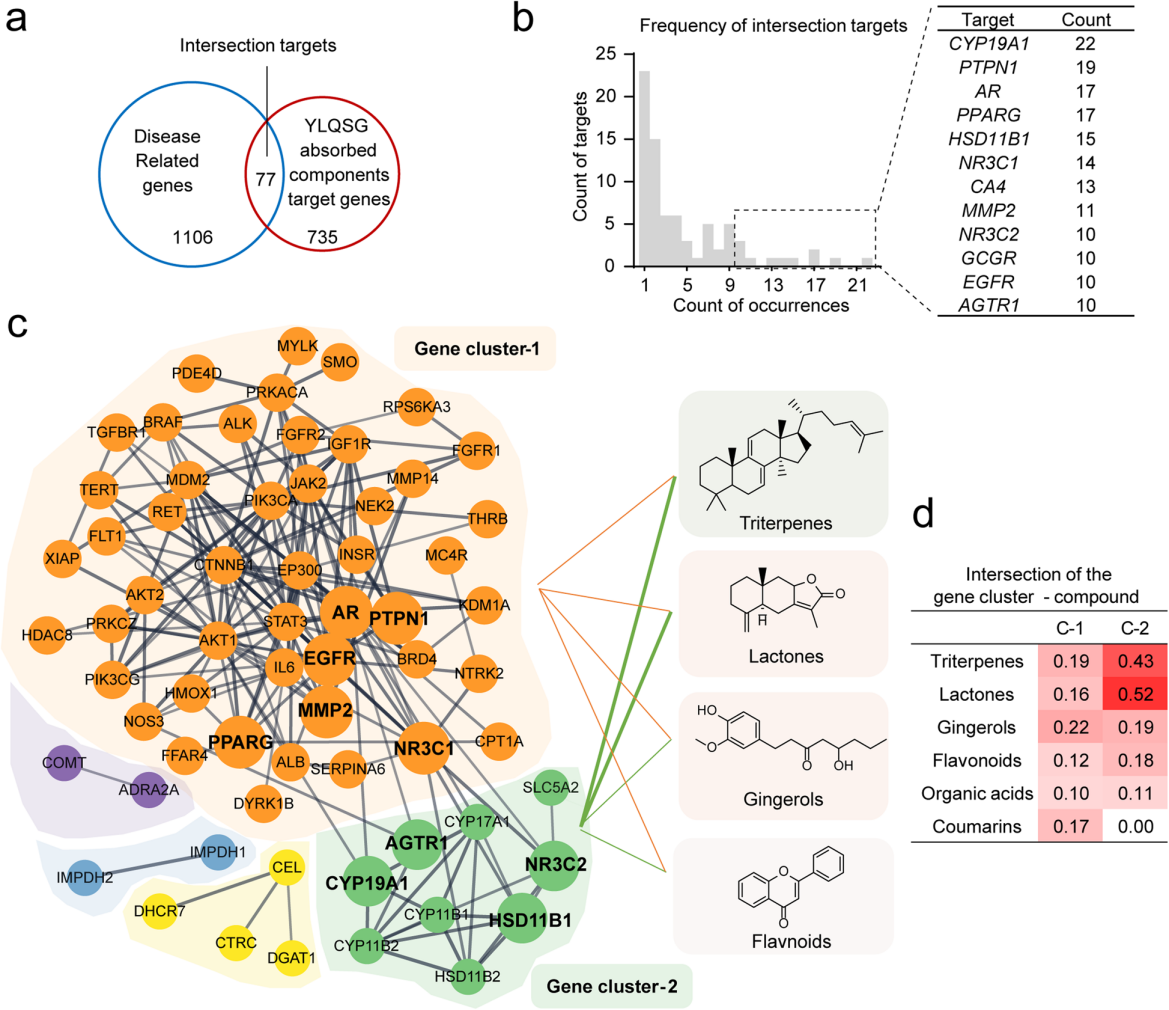
Target prediction of YLQSG prototypes in regulating glucose homeostasis and hypertension by Network Pharmacology (**a**) Venn diagram between MetS-associated genes and targets of absorbed prototypes from YLQSG. **b** High-frequencies targets. **c** STRING-based protein–protein interaction (PPI) network and functional clustering. The lining thickness was proportional to the association power between structural classes and gene clusters. **d** Degree of correlation of structural classes interacting with gene clusters

To explore how YLQSG's absorbed components might exert synergistic effects in treating MetS-related complications, such as hypertension, we performed network pharmacology. From the absorbed components of YLQSG, 735 structural based potential targets were proposed, 77 of which overlapped with MetS disease targets (Fig. 6a). The frequency of intersecting targets indicates strong protein–ligand interactions (Fig. 6b). Considering the interwoven interactions among proteins, A PPI network was constructed to identify node proteins with the most pharmacological relevance, revealing two major clusters: Cluster 1, associated with insulin homeostasis (INSR, PTPN1, GCGR, CTNNB1, STAT3), and Cluster 2, related to the renin–angiotensin–aldosterone system (RAAS) (AGTR1, NR3 C2, HSD11B1, HSD11B2) (Fig. 6c). Notably, cluster2 target genes were strongly correlated to lanosterol triterpenoids attributed to the sovereign herb *Poria cocos* (Schw.) Wolf (Fuling, FL) (Fig. 6d).

**Fig. 7:**
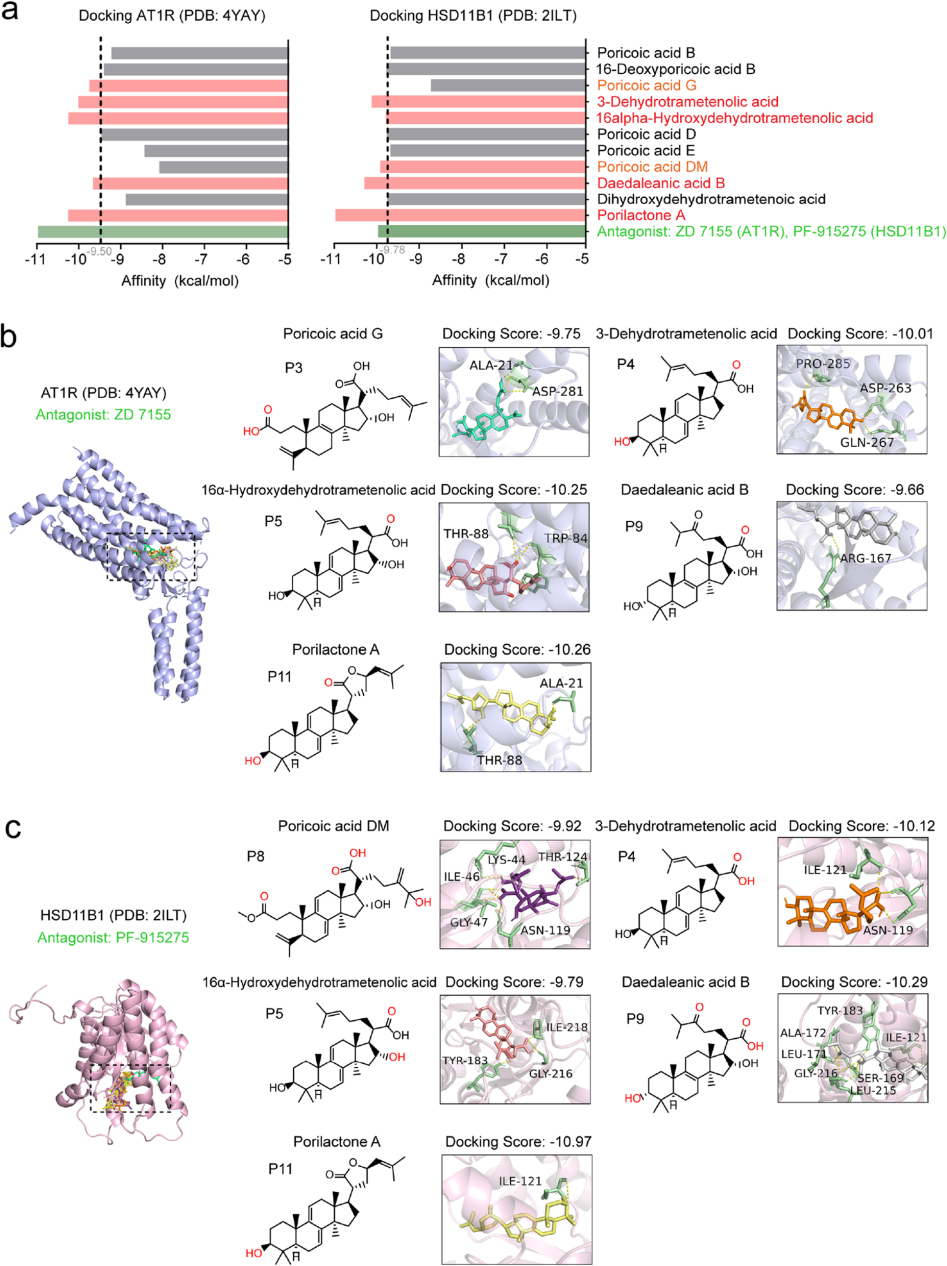
Molecular docking of triterpenoids in YLQSG (**a**) Molecular binding affinities of triterpenoids in YLQSG and known antagonists with key RAS proteins AT1R (PDB ID: 4YAY) and HSD11B1 (PDB ID: 2ILT). **b**-**c** Crystallographic binding poses of antagonists and triterpenoids. The protein structures are presented in light gray or pink cartoon. The residues lining, binding sites, and ligands are presented in capped sticks, as presented. Helices and residues lining pockets are labeled, and hydrogen bonds are displayed as black dashed lines

To elucidate the potential roles of triterpenoids in regulating RAAS via cluster2 genes, molecular docking of 11 absorbed triterpenoids with the most frequent targets AT1R and HSD11B1 were conducted, using ZD7155 (an AT1R antagonist) and PF- 915275 (a HSD11B1 antagonist) in comparison (Fig. 7a). For AT1R, 11 triterpenoids showed high binding affinities, with docking scores lower than − 8 kcal/mol, among which porilactone A exhibited the strongest (− 10.26 kcal/mol). Hydrogen bonds formed between carbonyl group on the side chain and residue Thr- 88, and between the hydroxyl group on A ring and residue Ala- 21, were observed in poricoic acid G and porilactone A (Fig. 7b). Similarly, docking with HSD11B1 revealed strong interactions of 5 triterpenoid ligands. For porilactone A, daedaleanic acid B and 3-dehydrotrametenolic acid, they even surpassed the binding affinity of well-recognized antagonist PF- 915275, which may partially contribute to hydrogen bond with Ile- 121 that help increasing the stabilities of both hydroxyl group on the A ring and carbonyl group on the side chain (Fig. 7c).

### Pharmacokinetics of absorbed triterpenoids of YLQSG in rat serum

**Fig. 8:**
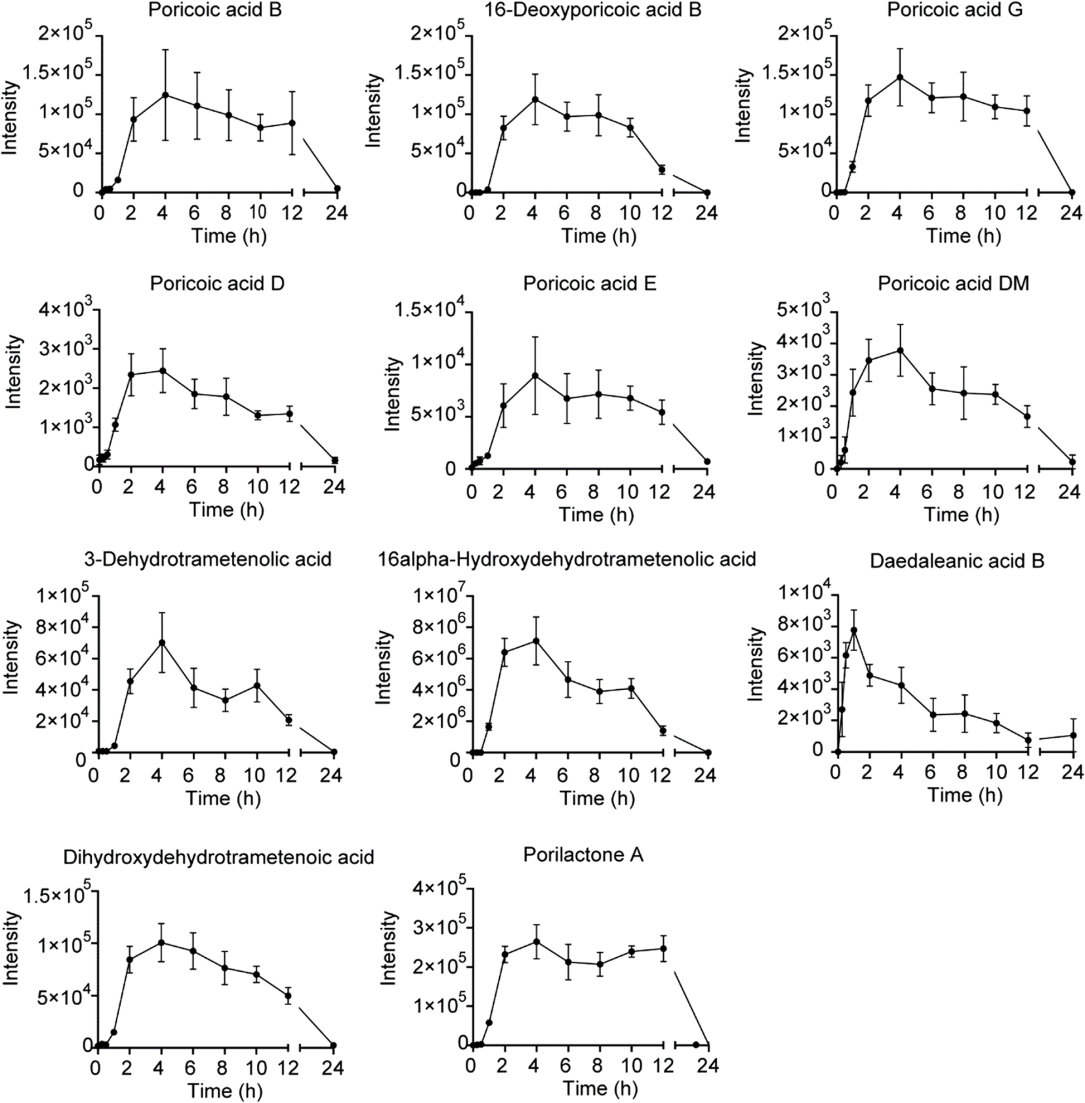
Serum concentration–time curve of 11 feature prototype compounds in SD rats after single dose of YLQS administration Data are presented as mean ± SEM (*n* = 6)

To further gain knowledge of the in vivo time-concentration dynamics of these proposed triterpenoid candidates, we explored the pharmacokinetics in rat serum (Fig. 4a). After single gavage of YLQSG, the blood-concentration curve of absorbed components was depicted (Fig. 8). Most compounds had a single-peak absorption, whereas 3-dehydrotrametenolic acid and porilactone A showed a double-peak absorption pattern, probably due to enterohepatic circulation or gastric emptying. Majority of absorbed prototypes had the maximum serum concentration (Cmax) within 5–8 h post administration, while daedalean acid B was absorbed more rapidly, with a Tmax of 1.35 ± 0.68 h (Table 3). As for elimination, all 11 prototypes showed rapid serum clearances with half-life (t1/2) of less than 7 h, suggesting a low risk of drug accumulation. Area under the curve (AUC0-t) statistics showed highest serum abundance of 16α-hydroxydehydrotrametenolic acid and porilactone A (Table 3). In line with molecular docking, these two triterpenoids were also among the strongest binding affinities (< − 9.78 kcal/mol) to blood pressure modulate targets AT1R and HSD11B1, emphasizing them as promising pharmacologically contributing drug candidates for mitigating not only DS but also MetS associated hypertension.

**Table 3:** Pharmacokinetic parameters of 11 feature prototypes after YLQS administration in SD rats (*n* = 6, mean ± SEM)

Parameters^a^	Cmax	Tmax	t1/2	AUC(0-t)	AUC(0-∞)
	μg/L	h	h	μg/L*h	μg/L*h
Poricoic acid B	213.6 ± 53.8	5.50 ± 0.96	4.11 ± 0.82	2709.0 ± 417.0	2292.8 ± 621.1
16-Deoxyporicoic acid B	290.3 ± 44.5	5.60 ± 0.75	4.95 ± 0.81	1966.4 ± 280.2	2535.1 ± 263.6
Poricoic acid G	469.5 ± 89.2	5.50 ± 0.96	3.50 ± 1.08	4089.5 ± 442.1	4423.6 ± 366.7
Poricoic acid D	7.5 ± 1.3	6.40 ± 1.72	5.51 ± 0.88	68.0 ± 10.1	82.0 ± 5.1
Poricoic acid E	16.0 ± 2.7	8.50 ± 1.50	6.64 ± 0.75	204.7 ± 25.1	222.9 ± 30.6
Poricoic acid DM	13.2 ± 2.0	4.00 ± 1.41	4.61 ± 1.21	77.1 ± 12.4	104.9 ± 13.6
3-Dehydrotrametenolic acid	187.6 ± 37.6	4.33 ± 0.33	3.33 ± 0.54	1325.4 ± 159.3	1434.5 ± 149.0
16alpha-Hydroxydehydrotrametenolic acid	16,750.2 ± 1950.9	4.40 ± 0.75	1.78 ± 0.09	135,885.1 ± 12,105.3	135,927.1 ± 12,100.3
Daedaleanic acid B	20.6 ± 0.8	1.35 ± 0.68	3.84 ± 0.75	129.0 ± 32.5	169.7 ± 27.6
Dihydroxydehydrotrametenoic acid	307.8 ± 35.6	4.40 ± 0.75	4.98 ± 1.07	3034.0 ± 355.9	3271.4 ± 141.5
Porilactone A	846.0 ± 31.9	6.50 ± 1.89	2.15 ± 0.24	9462.0 ± 500.6	9200.2 ± 744.0

^a^*C*_*max*_ the maximum serum concentration of the drug achieved within the body, *T*_*max*_. time to reach C_max_, *t*_*1/2*_ half washout time of the drug in the body, *AUC* Area under the serum concentration–time curve

## Discussion

For chronic metabolic diseases, such as MetS, one of the biggest challenges is the lack of long-term intervention with prominent efficacy and safety. In this study, we demonstrated that YLQSG, a well-recognized TCM prescription in treating DS, has promising beneficial effects on MetS-associated hypertension and obesity-related lipid and glucose metabolic disruptions, probably through regulating blood pressure by lanosterol triterpenoids in the sovereign herb *Poria cocos* (Schw.) Wolf (Fuling, FL) via targeting RAAS system. This suggests the potential of YLQSG in the arsenal for combination therapy of MetS that deserves future explorations, both mechanically and clinically.

MetS patients are often suffered from hypertension, with estimated prevalence rate from 20% to approaching 50% (Ford et al. 2002). Hypertension has been recognized by the World Health Organization (WHO) as a critical predictive risk factor for cardiovascular diseases, particularly in diabetes mellitus (Stamler et al. 1993). Some results showed that hypertension was associated with a 72% increase in the risk of all-cause mortality and a 57% increase in the risk of cardiovascular events (Chen et al. 2011). Targeted blood pressure (BP) control has been shown to reduce diabetes-related mortality, alleviate the progress of diabetic retinopathy (UKPDS 1998), and improve cardiovascular outcomes (Hansson et al. 1998; HOPE 2000). The TECOS Trial (Green et al. 2015, 2013), which evaluated secondary prevention interventions—including blood pressure control (systolic BP ≤ 140 mmHg; diastolic BP ≤ 90 mmHg)—among 13,616 diabetic patients from 38 countries, found a negative correlation between the degree of secondary prevention and the incidence of primary cardiovascular events (Pagidipati et al. 2017). Additionally, the ACCORD BP trial (Cushman et al. 2007) compared an intensive blood pressure intervention strategy (systolic BP ≤ 120 mmHg) to standard BP therapy (systolic BP ≤ 140 mmHg) among high-risk individuals with diabetes and found that intensive BP therapy can lead to a significant reduction in total and nonfatal stroke, reinforcing the need for targeted blood pressure control (Cushman et al. 2010). Despite these benefits, however, only one-third of patients received proper secondary prevention care, with blood pressure and LDL-C being the most poorly controlled factors (Pagidipati et al. 2017). In line with these studies, there is a growing consensus that blood pressure control should be prioritized in the management of diabetes and MetS to reduce the burden of severe complications. For long-term health benefits, the American Heart Association (AHA) and the National Heart, Lung, and Blood Institute (NHLBI) recommend maintaining blood pressure below 140/90 mmHg in patients with MetS and below 130/80 mmHg in those with diabetes (Grundy et al. 2005).

In TCM theory, MetS and its associated complications, such as hypertension, can be characterized by its unique causal endotypes, majority of which refers to as DS. Mechanistically, DS is frequently caused by spleen deficiency. In TCM, spleen is considered responsible for biofluid transportation and transformation in the body (Dou et al. 2021), so spleen deficiency would cause inefficient fluid metabolism, leading to dampness accumulation and potential metabolic disorders. Previous studies have highlighted the significant role of Dampness-Heat Syndrome—the most prevalent DS subtype—in hepatic diseases, including non-alcoholic fatty liver disease, chronic viral hepatitis, and liver cirrhosis (Dai et al. 2013; Guo et al. 2012; Jiang et al. 2021; Li et al. 2021; Wen et al. 2020). Beyond liver diseases, Zhou et al. (2024) reported a correlation between DS severity and serum triglyceride levels, suggesting its contribution to hyperlipidemia-related metabolic diseases (Zhou et al. 2024). Consistent with this, our results also showed a high prevalence of DS among MetS patients, majority of which had concomitant hypertension (88.68%, 47/53). Further phenotypic analysis revealed frequent signs of DS related to biofluid transport, such as an enlarged tongue, excessive salivation, and shapeless, pasty stools, suggesting a strong association between DS and MetS.

TCM has long been used for DS, not simply treat, but also cure. Herbs such as *Poria cocos* (Schw.) Wolf (Fuling), *Atractylodes macrocephala* Koidz. (Baizhu), *Polyporus umbellatus* (Pers.) Fries (Zhuling) are well-recognized and widely-applied in TCM for spleen-tonifying and dampness-repellin*g.* In particular, *Poria cocos* (Schw.) Wolf (Fuling) and *Atractylodes macrocephala* Koidz. (Baizhu) is a famous"herb-pair"widely used in DS, with synergistic effects that exemplify"great combination and artful application"of TCM theory. However, despite their profound effects on DS, the potentials of these herbs in treating DS associated disease are under-explored and under-appreciated. To this concern, we investigated the effects of YLQSG, a classical dampness-dispersing formula based on the Fuling-Baizhu herb pair, and found that a 12-week YLQSG intervention alleviated DS symptoms and effectively reduced blood pressure (SBP/DBP) in MetS patients. To the best of our knowledge, our findings suggest the potential benefits of targeting TCM syndromes to improve disease outcomes, bridging the gap between TCM intervention strategies for DS and metabolic diseases.

For both MetS and diabetes, blood pressure requires long-term intervention, from years to decades. The UKPDS-HDS study (HDSG 1994) underscored the importance of sustained blood-pressure control in achieving long-term benefits (Holman et al. 2008b). Specifically, patients with type 2 diabetes and hypertension underwent either tight or less-tight blood-pressure treatment for four years, followed by a 10-year follow-up period (Holman et al. 2008b). Differences in blood-pressure reduction between the groups diminished within two years after the trial ended. In line with blood-pressure, benefits for diabetes-related outcomes—including reduced diabetes-related mortality, stroke, and microvascular complications—were not sustained during the follow-up (Holman et al. 2008b; Parati et al. 2011). Hence, long-term blood-pressure control strategies with both high efficacy and high safety is of great demand. Currently available antihypertensive drugs including diuretics, angiotensin-converting enzyme inhibitors (ACEIs), β-blockers, angiotensin receptor blockers (ARBs), and calcium channel blockers (CCBs), are all associated with varying degrees of safety concerns (Gress et al. 2000). Alarmingly, elevated fasting blood glucose had been reported with the use of thiazide-type diuretic of hypertension (Barzilay et al. 2006; Gress et al. 2000). In contrast, TCM herbs used for dispersing dampness have demonstrated excellent safety. For instance, Fuling (the dried sclerotium of the fungus *Poria cocos***)** is an edible mushroom widely used as a dietary ingredient (Ríos 2011), highlighting its high safety ideal for long-term consumption. Balancing both safety and efficacy, studies have shown that Fuling can significantly alleviate hepatic steatosis and rescue disrupted lipid metabolism in *ob/ob* mice, potentially by modulating butyrate-producing gut microbiota (Sun et al. 2019). Echoed with this, in our study, MetS patients who underwent 12-weeks of YLQSG administration exhibited a significant rescue of obese related lipid and glucose disruption, with reduced BMI, serum TG, and surprisingly, decreased HbA1c. Since early glycemic control in T2D patients has been showed to provide sustained benefits (Gress et al. 2000; Holman et al. 2008a), it’s worth investigating whether YLQSG treatment can induce a similar “legacy effect” and ultimately contribute to long-term health improvements.

Composition determines activity. To elucidate the material basis and mechanisms of YLQSG, it is essential to analyze its chemical composition. To the best of our knowledge, our study is the first to comprehensively reveal the chemical characterization of YLQSG, including its chemical composition, absorbed prototype constituents, and in vivo metabolites, providing valuable insights into its bioactive components. To identify active compounds of YLQSG responsible for its beneficial effects, network pharmacology analysis was conducted, revealing three structural categories with the highest potential: triterpenoids, lactones, and gingerols. Among these, triterpenoids gained our special attention, as many were not only efficiently absorbed into the bloodstream with relatively high serum abundance but were also predominantly derived from the sovereign herb Fuling (*Poria cocos* (Schw.) Wolf). Of note, Fuling is recognized for its diverse bioactive chemical constituents, the most predominant being (1–3)-β-D-glucan polysaccharides (Ng et al. 2024; Sun 2014) and triterpenoids (Guo et al. 2024; H. Liu et al. 2024a, b; Wang et al. 2013). Triterpenoids, particularly lanosterol-type which include various acids such as poricoic acid and pachymic acid, are regarded as the primary active components responsible for the therapeutic effects of Fuling, ranging from anti-tumor (Cheng et al. 2013, 2015) to anti-inflammatory (Cai and Cai 2011; Gui et al. 2021) and anti-diabetic effects (Chen et al. 2019; Ding et al. 2024; Li et al. 2011). For its anti-diabetic effects, the total triterpenoid extract of Fuling has been shown to mitigate diabetic ulcers by promoting endothelial migration, inducing M2 macrophage polarization, and reducing focal inflammation (Ding et al. 2024). In another study, the triterpenoids dehydrotumulosic acid, dehydrotrametenolic acid, and pachymic acid from Fuling had been reported with insulin-sensitizing activities to varying degrees (Li et al. 2011). An in-silico structure–activity relationship analysis further suggested that the 6/6/6/5 ring skeleton and the double bond between C- 8 and C- 9 contribute to glucose uptake stimulation and such insulin-sensitizing activity (Chen et al. 2019). It is particularly noteworthy that two studies have investigated the effects of triterpenoids on biofluid regulation and blood pressure control. Either oral or intraperitoneal administration of Fuling triterpenoid extracts have been shown to alleviate inflammation-induced hind paw and ear edema (Giner-Larza et al. 2000). Gavage of triterpenoid poricoic acid A effectively reduced both systolic and diastolic blood pressures while improving renal functions in nephrectomy-induced rats, probably through modulation of the AMPK-Smad3 cascade (Chen et al. 2020). However, such effects are inadequately elucidated and the molecular targets have yet to be clearly identified.

Through target functional clustering of absorbed prototypes of YLQGS, we identified a strong correlation between triterpenoids and Cluster 2 target genes, which is associated with the regulation of the Renin–Angiotensin–Aldosterone System (RAAS)—a well-established target for hypertension. Cluster 2 includes clinically approved antihypertensive druggable targets within the RAAS, such as angiotensin II receptor blockers like losartan and valsartan, which target AGTR1, and aldosterone antagonists like spironolactone and eplerenone, which target NR3 C2. In our study, molecular docking analysis revealed that lanostane-type triterpenoids from Fuling had strong binding affinities with AGTR1 and 11β-HSD1. Since 11β-HSD1 has been proposed as a promising therapeutic target for metabolic diseases including diabetes mellitus and MetS (Gathercole et al. 2013; Tomlinson et al. 2004; Wamil and Seckl 2007), such results suggesting a potential synergistic effect of Fuling triterpenoids in RAAS regulation. Previous studies have reported oleanane-type specific triterpenoids, for example, the oleanolic acid, can bind to AGTR1 (T. Liu et al. 2024a, b). Similarly, oleanane-type 18β-glycyrrhetinic acid and its hemisuccinyl ester derivative, carbenoxolone, are reported as 11β-HSD1 inhibitors (Hult et al. 2006; Stewart et al. 1987). However, lanostane-type triterpenoids were highly underappreciated. Our results indicated that lanostane-type triterpenoids—such as porilactone A and 16α-hydroxydehydrotrametenolic acid—would act on as dual ligands for AGTR1 and 11β-HSD1, highlighting their great potential as novel antihypertensive agents with a distinct structural skeleton that deserves further exploration and validation.

Current research highlights the significant medicinal potential of triterpenoids (Goddard et al. 2024). So far as we know, four triterpenoids have been approved for clinical applications in treating diseases such as Friedreich’s ataxia, vulvovaginal candidiasis, and peptic or oral ulcers, alongside with over 50 registered clinical studies investigating triterpenoid-based therapies (Goddard et al. 2024). Although no triterpenoid has been applied in treating hypertension and MetS in clinical studies, our results propose lanostane-type triterpenoids from herb Fuling may serve as a promising lead compound library for hypertension treatment and the related works are planning to be carried out in our laboratory. To evaluate the drug likeness properties of these lanostane-type triterpenoids, we further plotted the drug serum concentration–time curves and calculated in vivo pharmacokinetic parameters. The plasma absorption and elimination kinetics suggested that these 11 triterpenoids exhibit favorable pharmacokinetic properties, indicating promising druglikeness in future drug development.

In summary, this study demonstrated that YLQSG, a Traditional Chinese Medicine (TCM) prescription for DS, holds promising therapeutic potential for MetS by alleviating hypertension and obesity-related metabolic disruptions. Through comprehensive characterization of YLQSG’s chemical composition, absorbed prototype constituents, in vivo metabolic transformation, and pharmacokinetics, we identified lanostane-type triterpenoids as promising lead compounds for targeting AGTR1 and 11β-HSD1. These findings highlight the potential of YLQSG in the arsenal for combination long-term therapy in managing MetS and its associated complications, such as hypertension and hyperlipidemia, warranting further investigation.

## Data Availability

The datasets during and/or analysed during the current study available from the corresponding author on reasonable request.
